# Complete Hearing Recovery after Retrosigmoid Resection of Jugular Foramen Schwannoma with Concurrent Ipsilateral Vestibular Schwannoma

**DOI:** 10.1055/a-2516-7311

**Published:** 2025-02-06

**Authors:** Achilles A. Kanaris, Nicholas E.F. Hac, Stephen T. Magill, Kevin Y. Zhan

**Affiliations:** 1 Northwestern University Feinberg School of Medicine, Chicago, Illinois, United States; 2 Department of Neurology, Northwestern University Feinberg School of Medicine, Chicago, Illinois, United States; 3 Department of Neurological Surgery, Northwestern University Feinberg School of Medicine, Chicago, Illinois, United States; 4 Department of Otolaryngology-Head and Neck Surgery, Northwestern University Feinberg School of Medicine, Chicago, Illinois, United States

**Keywords:** hearing recovery, hearing return, retrosigmoid, jugular foramen schwannoma

## Abstract

**Introduction** Cerebellopontine angle (CPA) tumors frequently present with hearing loss, which influences whether a hearing-preservation versus hearing ablative surgical approach is chosen. We discuss a case of complete hearing recovery after resection of a jugular foramen schwannoma (JFS) in a patient who also had a small intracanalicular vestibular schwannoma (VS).

**Case** A 46-year-old woman presented with left ear fullness, tinnitus, and imbalance for 9 months. She had no lower cranial nerve dysfunction. Audiometry demonstrated Class D hearing with 4% word recognition on the left. Vestibular testing showed absent caloric response on the left and subtle central findings. Magnetic resonance imaging demonstrated a left 3.3-cm JFS and separate left 1-cm intracanalicular VS. A retrosigmoid approach was performed for a radical subtotal resection of the JFS, relieving the mass effect on the vestibulocochlear nerve. The small intracanalicular VS was not manipulated. Pathology confirmed schwannoma with neurofibromatosis type 2 (NF2) mutation in the tumor but normal NF2 germline. Postoperative audiometry at 6 weeks showed normal audiometric thresholds with 100% discrimination. Subtle left caloric response was noted on postoperative vestibular testing and central oculomotor findings improved.

**Discussion** The presented case describes the management of concurrent ipsilateral VS and JFS in the absence of NF2 and demonstrates a unique complete and rapid recovery of hearing following JFS resection without manipulation of concurrent VS. This case supports the use of a hearing-preservation approach in similar cases and corroborates previous reports of hearing recovery following resection of non-VS CPA tumors with hearing-preservation approaches.

## Introduction

Cerebellopontine angle (CPA) tumors comprise multiple entities and represent 5 to 10% of all intracranial neoplasms. 1 Vestibular schwannomas (VSs) are the most common type of CPA tumor, accounting for about 80% of cases, whereas other cranial nerve (CN) schwannomas comprise roughly 2.7% of all tumors in the region. 1 2 3 Jugular foramen schwannomas (JFSs), which arise from CNs IX, X, and XI, represent an even smaller subset of this group, accounting for 2.9 to 4% of all intracranial schwanommas. 4 Tumors arising from the CPA may have overlapping presentations including unilateral disturbances to CN V, VII, and VIII with cerebellar dysfunction. 5 While JFSs can present with lower cranial nerve (LCN) dysfunction, unilateral sensorineural hearing loss (SNHL) is the most common presenting symptom of both VS as well as JFS. 6 7 Choice of surgical approach takes into account many aspects including tumor size, location, and extension, as well as symptoms at presentation—such as preoperative hearing. 4 7 8 9 10 11 The serviceability of preoperative hearing influences whether a hearing-preservation versus hearing ablative surgical approach is chosen. Hearing-preservation approaches such as the retrosigmoid approach may be chosen when preoperative hearing is serviceable, whereas hearing ablative approaches like the translabyrinthine approach may be selected in cases of severe preoperative hearing loss, especially when this allows a more favorable surgical corridor. 11 12

Outside of neurofibromatosis type 2 (NF2), multiple intracranial tumors are rare - even more so when isolated to within the CPA. 13 14 We discuss a unique case of complete and rapid hearing recovery after retrosigmoid resection of a JFS in a patient who also had a concurrent ipsilateral small intracanalicular VS, with implications for the management of similar cases.

## Case

**Fig. 1:**
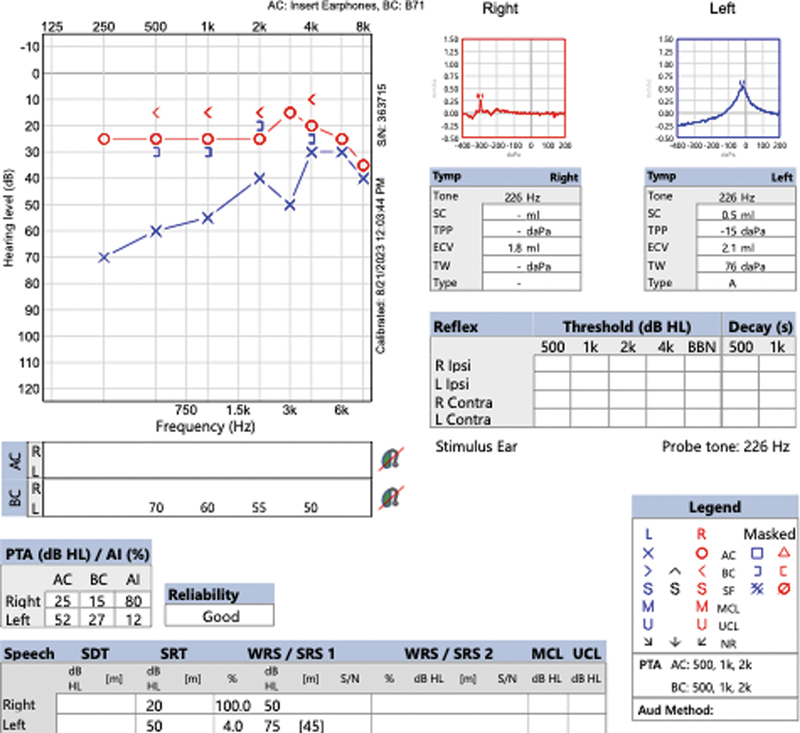
Preoperative audiometric testing showing significantly reduced pure tone thresholds and 4% word recognition in the affected ear, consistent with AAO-HNS class D hearing. AAO-HNS, American Academy of Otolaryngology—Head and Neck Surgery.

**Fig. 2:**
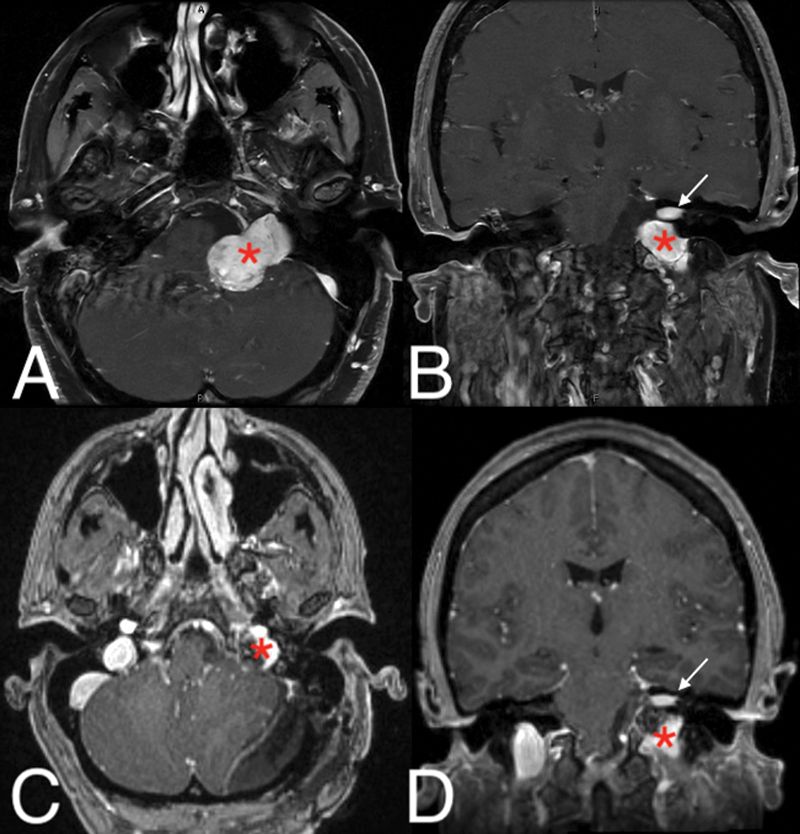
( **A, B** ) **.** Preoperative MRI with JF tumor (*) and separate JF (*) and IAC tumors (arrow). ( **C, D** ) **.** Postoperative MRI with residual JF tumor (*) and residual JF tumor (*) with nonmanipulated IAC tumors (arrow). IAC, internal auditory canal; JF, jugular foramen; MRI, magnetic resonance imaging.

**Fig. 3:**
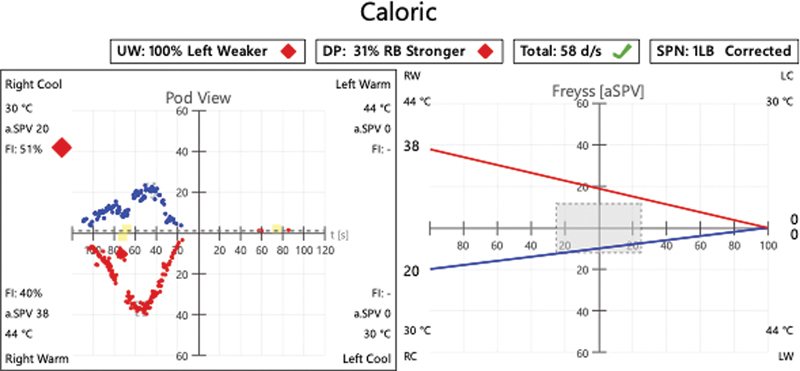
Preoperative caloric testing demonstrating completely absent response in the left ear, as no nystagmus was elicited with the application of either hot or cold water.

A 46-year-old woman presented to the Otolaryngology clinic with primarily left ear fullness. She reported tinnitus and imbalance for 9 months. She had no LCN dysfunction on clinical exam and flexible laryngoscopy. Audiometry showed significantly reduced pure tone thresholds and 4% word recognition in the affected ear, consistent with American Academy of Otolaryngology—Head and Neck Surgery (AAO-HNS) class D hearing ( Fig. 1 ). Weber lateralized to the right ear and clinical suspicion was high for a sensorineural hearing loss. Magnetic Resonance Imaging (MRI) demonstrated a large left 3.3-cm JFS and separate left 1-cm intracanalicular VS extending to the internal auditory canal (IAC) fundus, without an appreciable fundal cap ( Fig. 2A,B ). The jugular foramen tumor had a bilobed dumbbell appearance and extended from the jugular foramen to within the left CPA cistern. The tumor was causing brainstem compression as well as midline shift. Preoperative vestibular testing showed absent caloric response on the left ( Fig. 3 ) as well as numerous central ocular motor deficits including spontaneous left-beat and downbeat nystagmus, gaze-evoked nystagmus, impaired smooth pursuit, optokinetic nystagmus, and a central positional nystagmus.

**Fig. 4:**
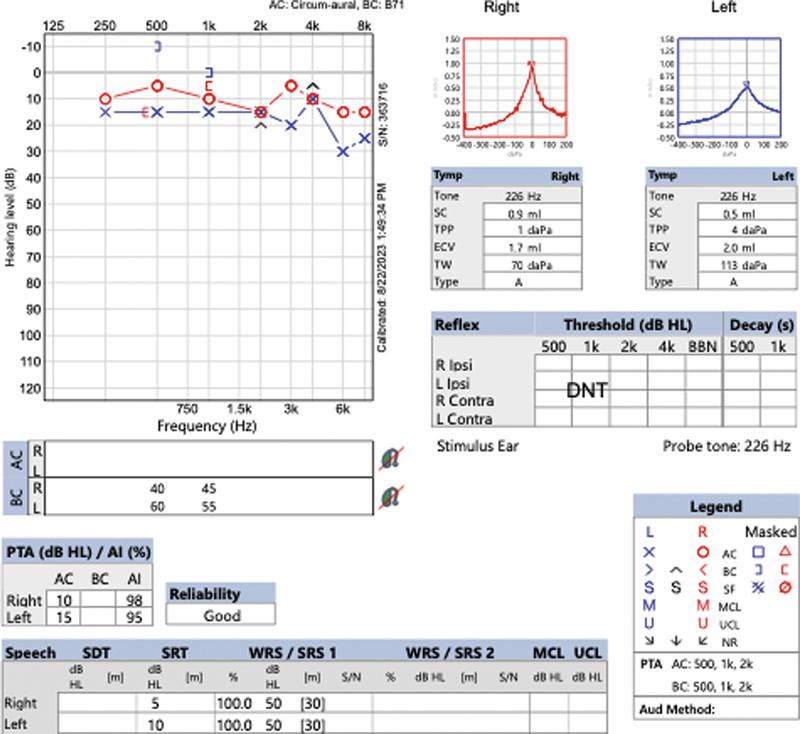
Postoperative audiometric testing showing normal audiometric thresholds with 100% discrimination, consistent with AAO-HNS class A hearing. AAO-HNS, American Academy of Otolaryngology—Head and Neck Surgery.

**Fig. 5:**
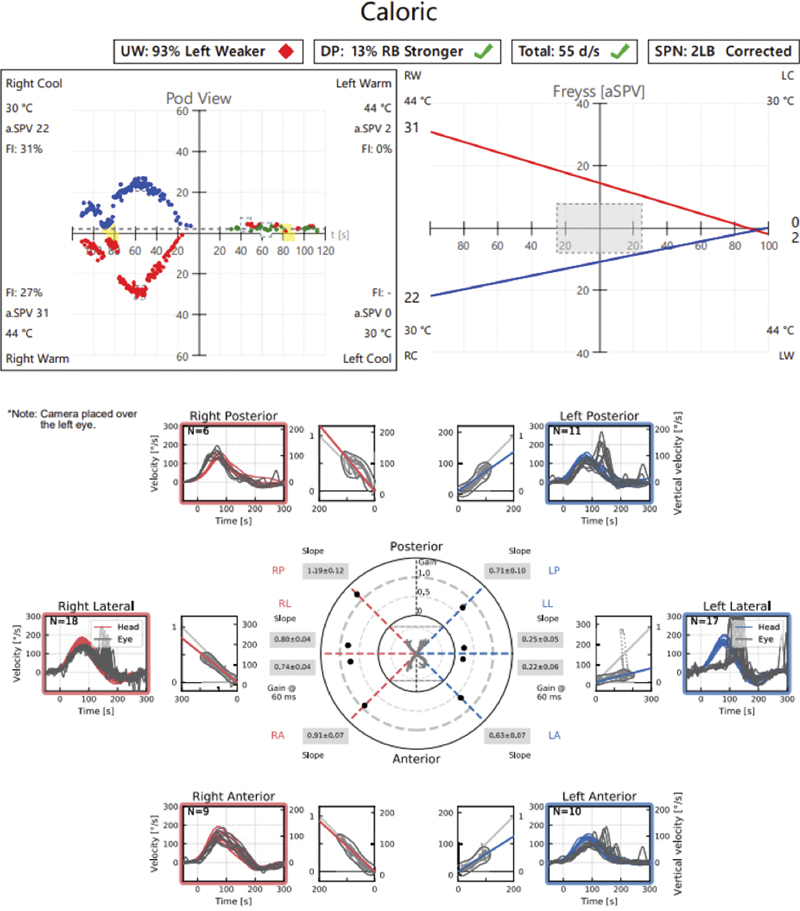
Postoperative caloric testing and video Head Impulse Test (vHIT) demonstrating a subtle response in the left ear, as a very weak nystagmus was elicited with both hot and cold water. The above vHIT demonstrates significant corrective saccades with loss of gain in the superior vestibular nerve > inferior vestibular nerve, demonstrating ongoing unilateral vestibulopathy.

A retrosigmoid approach was selected with the goal of removing only the JFS, which was found to be impinging on the vestibulocochlear nerve. Both tumors were clearly separate and the inferior trough of the IAC was removed to create access to the JFS. A radical subtotal resection of the JFS was achieved, relieving the stretch on the vestibulocochlear nerve and brainstem compression. Drilling of the posterior petrous face provided additional access to the tumor in the enlarged jugular foramen. An endoscope was used through the retrosigmoid approach to resect additional lateral and inferior components of the tumor. A small rind of tumor was left behind, which was tethered to the LCNs. The small intracanalicular VS was not manipulated and the IAC dura was left intact ( Fig. 2C,D ). Pathology showed a World Health Organization grade 1 schwannoma and next-generation sequencing identified an NF2 mutation in the tumor. Germline testing for NF2 was negative, confirming somatic NF2 mutation only in the tumor. The patient had significant improvement in postoperative hearing on postoperative day 1 and Weber tuning fork exam lateralized to the operated ear. Postoperative audiometry at 6 weeks showed normal audiometric thresholds with 100% discrimination (AAO-HNS class A hearing) ( Fig. 4 ). Peripheral vestibulopathy was relatively unchanged on postoperative vestibular testing but central findings resolved ( Fig. 5 ). MRI at 6-month follow-up demonstrated no growth of either tumor or no balance deficits. The patient will be followed with serial imaging, with consideration of radiosurgery to the small IAC tumor and residual jugular foramen tumor in the future.

## Discussion

The presence of multiple intracranial tumors, let alone separate ipsilateral schwannomas, is an incredibly rare occurrence outside of NF2. To date, there is only one other case report of concurrent ipsilateral VS and JFS without germline NF2 mutations reported in the literature. 15 That patient had a progressive unilateral SNHL that improved postoperatively. However, this was following resection of both the VS and JFS rather than the JFS alone as was seen in our case. This highlights the unique nature of the presented case as only the JFS in our patient was causing hearing loss. While we cannot discern whether the persistent unilateral vestibular loss was due to irreversible stretch injury from the JFS or from the intracanalicular VS, the mild recovery of vestibular function suggests the JFS was responsible for some of the initial vestibular loss.

While the underlying mechanism of rapid reversible hearing loss has yet to be fully understood, we theorize that the preoperative hearing loss was due to stretching of the junction between the vestibulocochlear nerve and its entry point to the brainstem or from compression of the fascicle within the brainstem. Subsequent resection of the jugular foramen tumor likely resolved this stretch and compression, leading to a complete and rapid resolution of the patient's severe SNHL with modest improvement in low-frequency caloric vestibular testing. It is remarkable that this recovery occurred without manipulation of the ipsilateral VS. There have been many similar cases of reversible hearing loss following resection of non-VS CPA tumors reported in the literature, from epidermoid tumors to meningioma and JFS. 16 17 18 19 20 21 22 23 24 These reports have proposed mechanisms for the observed reversible hearing losses, frequently citing improvements in neuropraxia or compression neuropathy following resection of tumor, leading to improved blood flow and offloaded pressure and stretch on the vestibulocochlear nerve. Notably, these improvements may occur over the course of a few days or weeks after the operation, potentially even in the first few postoperative days as with our patient. 16 17 Taken together, these factors highlight how non-VS CPA tumors may have different etiologies and prognoses as compared with VS with regard to hearing, with complete and even rapid recovery of hearing possible postoperatively. 17 23 24

Indeed, for JFS specifically, several authors have advocated for the avoidance of hearing ablative approaches, citing the possibility of hearing improvement following JFS resection. 4 10 25 The results of this report further substantiate this argument and add to the growing body of evidence that recovery of hearing loss is possible with resection of non-VS CPA tumors.

Overall, there is no current consensus for optimal surgical management of JFSs. 7 8 26 Samii et al offered a classification of JFS to help guide the selection of surgical approach for resection, building on the classification scheme previously proposed by them. 4 10 However, much uncertainty remains around the optimal treatment for JFSs. This is in part due to concerns about CN injury from various surgical approaches. 8 LCN deficits are the most common complication following JFS resection, with reported rates of 18.6%, followed by transient facial nerve paresis in 13.0% of patients seen in the largest review of cases. 7 As such, intraoperative neuroelectrophysiological monitoring is critical to reduce potential postoperative complications. 7 8

Ultimately, the presented case supports the use of a hearing-preservation approach in similar cases, regardless of preoperative hearing status. Hearing ablative approaches, such as a translabyrinthine approach that we considered in this case, should be avoided in the management of JFSs, given the potential for hearing recovery, as this case exemplifies. Our findings corroborate previous reports of reversal of hearing impairment following resection of non-VS CPA tumors, including but not limited to JFSs, with hearing-preservation approaches.

## Conclusion

The presented case describes the management of concurrent ipsilateral VS and JFS in the absence of NF2 and demonstrates a unique complete and rapid recovery of hearing following JFS resection without manipulation of concurrent VS. This experience supports the use of a hearing-preservation surgical approach in the treatment of JFS even in cases of nonserviceable preoperative hearing, as hearing recovery is possible. These findings further substantiate previous reports of hearing recovery in both JFSs as well as other non-VS CPA tumors with hearing-preservation approaches.
